# Impact of Timing and Dosage of a Fluoroquinolone Treatment on the Microbiological, Pathological, and Clinical Outcomes of Calves Challenged with *Mannheimia haemolytica*

**DOI:** 10.3389/fmicb.2016.00237

**Published:** 2016-03-02

**Authors:** Guillaume Lhermie, Aude A. Ferran, Sébastien Assié, Hervé Cassard, Farid El Garch, Marc Schneider, Frédérique Woerhlé, Diane Pacalin, Maxence Delverdier, Alain Bousquet-Mélou, Gilles Meyer

**Affiliations:** ^1^Vetoquinol Global Drug DevelopmentLure, France; ^2^Ecole Nationale Vétérinaire de Toulouse, Institut National Polytechnique de Toulouse, Université de ToulouseToulouse, France; ^3^LUNAM Université, Oniris, UMR BioEpARNantes, France; ^4^Institut National de la Recherche Agronomique, UMR1331 ToxAlimToulouse, France; ^5^Institut National de la Recherche Agronomique, UMR1300 BioEpARNantes, France; ^6^Institut National de la Recherche Agronomique, UMR1225 IHAPToulouse, France

**Keywords:** early treatment, fluoroquinolone, decreased regimen, bovine model, *Pasteurellaceae*

## Abstract

The efficacy of an early and low inoculum-adjusted marbofloxacin treatment was evaluated on microbiological and clinical outcomes in calves infected with 4.10^7^ CFU of *Mannheimia haemolytica* A1. Twenty-two calves were included based on their rectal temperature rise in the 10 h after challenge and allocated in four groups, receiving a single intramuscular injection of saline (CON), 2 mg/kg marbofloxacin 2–4 h after inclusion (early treatment, E2), 2 or 10 mg/kg marbofloxacin 35–39 h after inclusion (late treatments, L2, L10). In CON calves, *M. haemolytica* DNA loads in bronchoalveolar lavages continuously increased from inclusion to day 4, and were associated with persistent respiratory clinical signs and lung lesions. At times of early and late treatments, *M. haemolytica* loads ranged within 3.5–4 and 5.5–6 log_10_ DNA copies/mL, respectively. Early 2 mg/kg marbofloxacin treatment led to rapid and total elimination of bacteria in all calves. The late treatments induced a reduction of bacterial loads, but 3 of 6 L2 and 1 of 6 L10 calves were still positive for *M. haemolytica* at day 4. Except for CON calves, all animals exhibited clinical improvement within 24 h after treatment. However, early 2 mg/kg treatment was more efficacious to prevent pulmonary lesions, as indicated by the reduction of the extension and severity of gross lesions and by the histopathological scores. These results demonstrated for the first time that a reduced antibiotic regimen given at an early stage of the disease and targeting a low bacterial load could be efficacious in a natural bovine model of pneumonia.

## Introduction

Bovine respiratory disease (BRD) is a major economic concern in young beef cattle production, with a huge economic impact on the farming industry due to production losses and disease treatment and prevention (United State Department of Agriculture, 2013). BRD is mostly multifactorial due to complex interactions between pathogens (virus and/or bacteria and/or fungi), animal (host) factors and non-infectious environmental variables, such as farm management determinants (Fulton, 2009). Among the Gram-negative bacteria, the virulence factors expressed by *Mannheimia haemolytica*, such as leukotoxin, make it the major bacterial agent of BRD, resulting in high mortality, and loss of productivity in young calves (Van Donkersgoed et al., 1993; Zecchinon et al., 2005). At the herd level, the disease prevalence in suckler calves or young bull production is estimated to be ~15%, with high variability within herds (Cusack et al., 2003; Assie et al., 2009; Pardon et al., 2011). Recently, *M. haemolytica* was detected by real-time PCR in France in ~20% of respiratory samples from BRD (Pelletier, 2013).

Because of the high prevalence of Gram-negative bacteria as involved pathogens, the frequent association between bacteria and viruses, and the absence of pathogen identification in field conditions, the control of BRD generally includes the use of Gram-negative spectrum antibiotics (Apley, 1997; Fulton, 2009). Two therapeutic strategies are commonly implemented by veterinarians and farmers. Metaphylaxis represents a current practice of early BRD treatment, that consists in treating the entire cohort of animals, in which only a few express clinical signs of BRD (Nickell and White, 2010; Forbes et al., 2011). The advantages of this approach are good survival rate in the cohort and ease of use (Nickell and White, 2010); it also has the disadvantage of exposing to antibiotics a potentially large proportion of animals intended for human consumption even when not required. A second therapeutic approach consists in treating only the affected animals, regardless of the severity of their clinical signs (O'Connor et al., 2013; Heins et al., 2014). This approach limits exposure to antibiotics and decreases the risk of developing resistant strains. However, it is usually seen as the “late approach” because the clinical signs and possibly extensive pulmonary damage have had time to develop prior to treatment, as demonstrated for *Pasteurellaceae* (Ackermann and Brogden, 2000; Dagleish et al., 2010). Fluoroquinolones, such as marbofloxacin, represent a frequent “late approach” treatment for BRD in Europe. Considering the uncertainty regarding the causal agent and time of disease onset, labeled doses are determined by pharmacokinetics (PK) and pharmacodynamics (PD) and dose determination experiments. These doses generally target a cluster of pathogens and consider the highest minimal inhibiting concentration (MIC) reported (Toutain et al., 2002; Valle et al., 2012). These conditions also lead to routine use of a “high” antibiotic dose.

Antimicrobial usage leads to unavoidable selection of resistance in pathogens and commensal bacteria (Courvalin, 2005, 2008). Previous evidence has demonstrated the transfer of resistant bacteria or genetic determinants of resistance between animals and humans (Aarestrup and Wegener, 1999; Angulo et al., 2004; Bywater, 2004). Therefore, a wide consensus in both human and veterinary medicine encourages the reduction of antimicrobial usage to reduce its potential impact on public health. In parallel recent studies have stressed the interests of early therapeutic interventions, when the infectious bacterial load is low, suggesting that the dose of antibiotics may be modulated concurrently with the infectious bacterial load in both human and veterinary medicine (Ferran et al., 2007, 2011; Martinez et al., 2012; Vasseur et al., 2014). For example, in an experimental mouse-lung infection model with *Pasteurella multocida*, a lower dosage of fluoroquinolone was required to achieve similar bacteriological outcomes when administered “early” compared with a late administration pattern (Ferran et al., 2011). Further, an *in vitro* study observed high bactericidal activity of marbofloxacin even at low concentrations on low inoculum of both *M. haemolytica* and *P. multocida* (Illambas et al., 2013). Up to now, such observations have not been reported in a natural host model. These findings open new perspectives regarding the rational use of antibiotics in farm animals, combining the objectives of reducing their consumption and preserving animal health. In this context, this study aimed to determine the impact of an early and decreased regimen of marbofloxacin on the bacteriological burden, the extent of pulmonary lesions and the clinical recovery in calves experimentally infected with *M. haemolytica* A1.

## Materials and methods

### Bacteria and culture

*M. haemolytica* A1 strain 11-01-840 was isolated from the lungs of a calf with clinical signs of BRD in 2010 and stored at −80°C. Prior to each use, one aliquot of the strain was defrosted, and cultured on a Mueller Hinton Agar plate. The MIC was determined in Mueller Hinton Broth in triplicate according to the CLSI reference methods (CLSI, 2009). The MIC of marbofloxacin was 0.03 μg/mL.

*M. haemolytica* A1 strain 11-01-840 inoculum was prepared as follows: at day-1 (D-1) prior to inoculation, the strain was incubated overnight in 500 mL of brain heart infusion broth (BHIB) at 37°C; at day 0 (D0) just prior to inoculation, 50 mL of BHIB was centrifuged at 3000 g for 20 min; pellets were collected in 10 mL of 0.9% NaCl and washed twice in the same buffer; and the final pellet was then diluted into pre-warmed 0.9% NaCl to obtain a final suspension containing 2.10^5^ CFU/mL. Each calf was inoculated with 200 mL of the final suspension.

### Experimental infection of calves with *M. haemolytica*

Animal experimentation was performed as prescribed by the guidelines of the European Community Council on Animal Care (2010/63/EU), in accordance with accepted human standards of animal care, under ethical agreement number TOXCOM/0021/AF GL (French Ministry of Agriculture, Ethics Committee -C2EA-86).

Thirty-two dairy Holstein and Normand calves were selected at birth and reared until to 2–3 weeks of age (weight ranged from 47 to 75 kg) in French experimental units with no history of BRD (INRA, Domaine de Borculo, Exmes, France). Calves were colostrum-deprived after birth and fed with an oral rehydration solution the first 2 days to avoid transfer of immunoglobulins against bovine respiratory syncytial virus (BRSV), bovine parainfluenza virus type (BPI3) as well as *M. haemolytica*. Then they were fed until day 7 of age with milk replacer and a colostrum substitute (negative for BRSV/BPI3 antibodies), as previously described (Riffault et al., 2010). Calves were transported at 2–3 weeks of age and allocated to specific experimental units in collective pens of three to four animals with free access to hay and fresh water (experimental units of Toulouse, France). They were fed *ad libitum* with starter food (Passio Floc Junior, SudOuestAliments, Anan, France) and once a day with a milk replacer (Laitine Tech®, Bonilait-protéines, Chasseuneuil du Poitou, France). Upon arrival at the experimental unit, calves received a single intramuscularly injection of E-vitamin and selenium (0.025 mg/kg, Selephérol®, Vetoquinol, France) and underwent a complete physical examination. They remained fully healthy during the 8-day period prior to challenge. Absence of bovine viral diarrhea virus (BVDV) in calves was assessed at birth by negative real-time RT-PCR (Taqvet-BVDV kit, Lifetechnologie). Absence of BRSV and BPI3 was subsequently confirmed by negative serology of calves (LsiVet bovine RSV serum, Life technologies) and negative quantitative RT-PCR (Taqvet BRSV-bPI3 kit, Life technologies) on bronchoalveolar lavages (BAL) at D0 just prior to challenge with *M. haemolytica*. Absence of *M. haemolytica* asymptomatic carriers was confirmed by negative qPCR (TaqVet™ Triplex *P. multocida and M. haemolytica*, Life technologies, USA) in nasal swabs 2 days prior to inoculation.

Challenge was performed in calves between 3.5 and 5.5 weeks of age by intratracheal route as previously described (Riffault et al., 2010). Briefly, calves were restrained (standing up); an area of 20 cm^2^ was shaved at the median part of the neckline. Then, a 18 G catheter (Intraflon G18, Vycon) was inserted between two tracheal rings ~10 cm below the larynx. After location verification, 200 ml of pre-warmed solution containing 2.10^5^ CFU/mL *M. haemolytica* was injected into the catheter, corresponding to a total of 4.10^7^ CFU per calf. Calves were allocated into four groups based on elevation of rectal temperatures after challenge. The basal temperature of each calf was calculated as the mean rectal temperature collected individually twice daily for the 3 days prior to challenge. After challenge, rectal temperatures were taken every 3 h for a period of 10 h.

During this 10-h period, 22 of 32 calves were included at time 0 (T0); their rectal temperature exceeded +1°C their previously basal temperature. Calves were randomly allocated to one of four groups. The control group (4 CON calves) received no antibiotic treatment; the E2 group (6 E2 calves) received an early single intramuscular injection in the neck of 2 mg/kg marbofloxacin (Forcyl®, Vetoquinol, Lure, France) at T1, 0–4 h after inclusion (T0). The L2 and L10 groups (6 L2 and L10 calves per group) were treated later at T2, at 35–39 h post-inclusion T0 (33–35 h later than the early treatment), with a single injection of 2 mg/kg or 10 mg/kg marbofloxacin, respectively.

**Figure 1:**
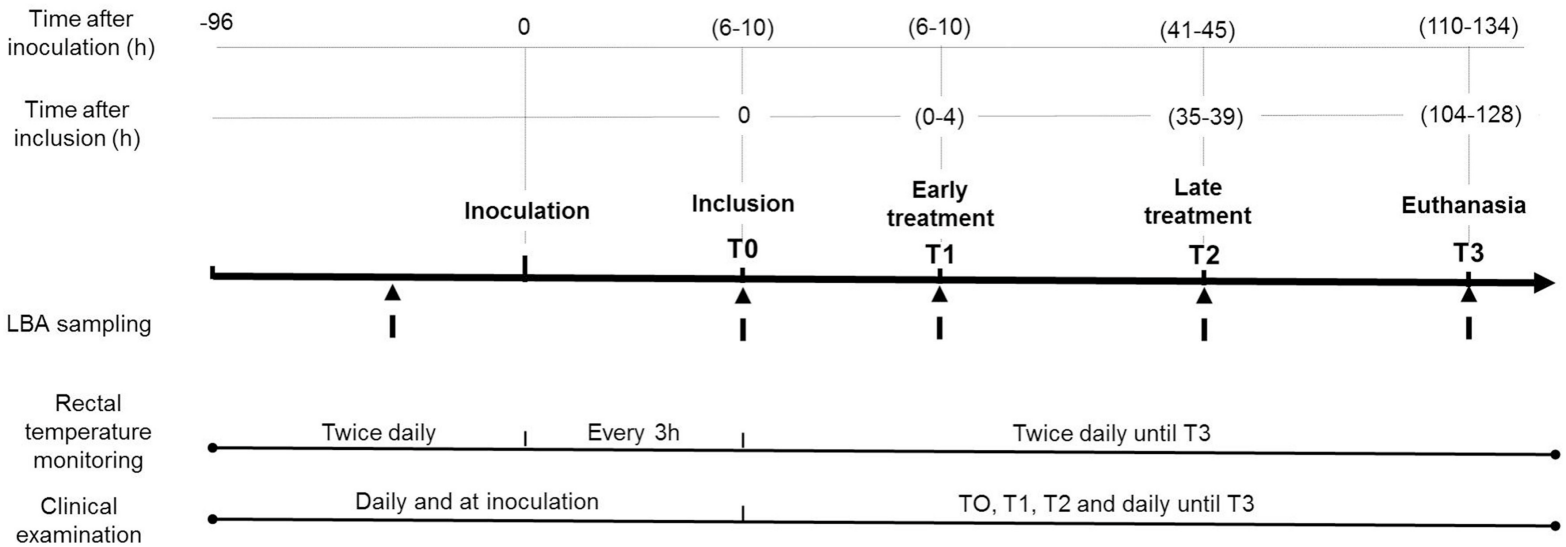
**Experimental timeline design of the study**.

To summarize, E2 calves (early treatment) were treated between 0 and 4 h and L2 and L10 calves (late treatment) between 35 and 39 h post-inclusion, corresponding to 6–10 and 43–48 h after *M. haemolytica* inoculation. Ten calves were excluded because they failed to develop hyperthermia (mean +1°C) in the 10-h period after inoculation of *M. haemolytica*. A description of the experimental design, chronology of sampling and clinical follow-up is presented in Figure 1.

The experiment ended 6 days after challenge, between 110 and 134 h post-challenge (time T3). Calves were euthanized during experimentation for ethical reasons or at T3 under general anesthesia overdose (5 mg/kg ketamine, followed by 15 mg/kg pentobarbital sodium and exsanguination).

### Clinical examination and respiratory sampling

Clinical observations were recorded twice a day from 3 days prior to the end of experimentation, between 110 and 134 h (T3) post-challenge. Rectal temperatures were measured every 3 h for a period of 10 h after challenge, then twice daily, and at times of inclusion (T0) and early (T1), and late (T2) treatments.

Calves were monitored using a scoring system adapted from Dowling et al. (2002). Scores were assigned as follow: demeanor from 0 to 3 (normal, dull, depressed, or recumbent); rectal temperature from 0 to 2 (<39.5°C, 39.5–40.5°C, >40.5°C); respiratory rate from 0 to 3 (<45, 45–60, 60–90, >90); heart rate from 0 to 2 (<80, 80–100, >100); nasal discharge from 0 to 3 (absent, mild, moderate, or profuse); intensity and added lung sounds from 0 to 2 (normal, increased effort, or labored); and appetite from 0 to 2 (normal, decreased, or anorexic). The clinical score was calculated daily for each calf by adding the values recorded for each parameter. In addition, individual accumulated clinical scores (ACS) were calculated for each calf as the area under daily clinical scores using the trapezoid method.

Bronchoalveolar lavages (BAL) were performed between 12 and 6 h prior to challenge for all 32 calves and at the end of the experiment for the 22 included calves. In addition, for three calves of each group (CON, E2, L2, and L10), BALs were performed just prior to T1 and T2, the times of early and late treatments, respectively. BALs on living animals were performed under general anesthesia (10 mg/kg ketamine and 10 mg/kg diazepam) by endoscopy (Olympus®, CF-EL) as previously described (Deplanche et al., 2007). For each BAL, a same volume of 120 mL of a sterile isotonic sodium chloride solution was injected into the same location of the right lobe. The collected volume varied between 65 and 80 ml per calf and analyses were carried out on 1 mL for each BAL. Titers were expressed by mL of BAL. BALs after euthanasia were performed by direct extraction and lavages of the lungs as previously described (Riffault et al., 2010). The recovered suspension was collected and stored at −80°C directly or after mixing with TRIzol® (Life Technologies, Saint Aubin, France) for quantitative *M. haemolytica* PCR analysis.

### Necropsy and tissue sampling

After euthanasia of the calves, gross lesions were photographed and assessed by visual examination and palpation of each of the pulmonary lobes. Moreover, the percentage of cranioventral lung consolidation was calculated as described by Amrine et al. (2014), as cranial and ventral lobes are the most common lesion sites.

Post-mortem samples were collected for histopathology and bacteriology, including cranial right and left, accessory, cardiac, and caudal left and right lobes of the lungs, as well as the mediastinal and tracheobronchial lymph nodes. For histopathology, tissue samples were paraffin wax-embedded after fixation in 10% neutral formalin, sectioned at 3–5 μm and stained with hemalun and eosin. A pathologist described and scored the severity of histopathology and inflammation in each slide as either normal (0), mild to moderate (1), or marked (2). A mild to moderate score of 1 was defined by fibrinous alveolitis with macrophagic infiltration, moderate quantities of mucus in the lumen of bronchioles and occasional few, small abbesses. A marked score of 2 was defined by hemorrhagic alveolitis with major leucocyte infiltration, alteration of neutrophils, foci of necrosis surrounded by oat cells, interlobular edema and thrombosis, moderate quantities of mucus in the lumen of bronchioles, and leucofibrinous pleuresia. A score of histopathological severity was calculated for each calf as the sum of all tissue slide-scores for that animal.

### *M. haemolytica* quantification

*M. haemolytica* quantification was performed by bacterial titration on post-mortem samples and quantitative PCR on BAL and post-mortem samples of included calves.

For titration, 1 g of the tissue sample collected from the right cranial lobe of the lung was homogenized in 9 mL of 0.9% NaCl. The homogenates were centrifuged at 3000 g for 10 min. The supernatants were discarded, and the pellets were resuspended in 10 mL of 0.9% NaCl. Ten microliters of successive 10-fold dilutions were then plated in triplicate on MH drug-free agar plates for incubation at 37°C. For all samples, bacterial counts were recorded after 24 h of incubation, and the lowest level of detection of colony counts was set at 100 CFU/g of tissue.

For quantitative PCR, DNA was extracted in elution buffer from 200 μL of BAL or 100 mg of tissue samples using a Macherey–Nagel nucleospin tissue genomic DNA kit. Five tissue samples were collected at the same location for each calf, including the right and left cranial, the accessory and the cardiac lobes of the lungs and the tracheobronchial lymphatic node. PCR was conducted with 5 μL of elution buffer (50 μL) with the TaqVet™ Triplex *P. multocida and M. haemolytica* kit (Life technologies, USA) according the manufacturer's instructions in a Light Cycler 480 (Roche). A standard plasmid curve for quantification was obtained by successive 10-fold dilutions of a plasmid containing 10^6^ DNA copies of the same *M. haemolytica* amplified nucleotide sequence (Life technologies, USA). In addition, two housekeeping genes previously mentioned in the literature (*gap*dh and *rpl19*) were amplified in each sample to normalize the qPCR products using GeNorm software (Biogazelle, qBase+). PCR results were analyzed using Roche Light Cycler 480 software (release 1.5.1) using the standard 2nd derivative and fit points methods for all samples (Roche Diagnostics). Results were similar when the PCR threshold was defined by the software (2nd derivative method) or fixed manually (fit points method) around 100 DNA copies. Results are expressed as the mean of PCR titers for each group and each day, after a logistic transformation, or as the accumulated bacterial shed (ABS), which corresponds to the mean area under individual curves of *M. haemolytica* detected by qPCR in BALs from T0 to T3 (GraphPad Prism software analysis, La Jolla, California, USA).

### Statistical analysis

Statistical analyses were performed using GraphPad Prism software (RRID: rid_000081, La Jolla, USA). For DNA quantification statistical analyses were performed on logarithmic-transformed data. A two-way ANOVA with repeated measures was used for DNA quantification in BAL and for clinical scores. The two-way ANOVA gave a *p*-value < 0.0001 for interaction between time/group (23.7 and 17.1% of total variation respectively). Therefore, we considered that the study had sufficient power for further analyses. As effects of the “day” and “treatment” factors were significant among interactions, Bonferroni adjustments for multiplicity were used between contrasts to compare the treatments on each day. A two-way ANOVA without repeated measures was used for DNA quantification in respiratory tissues. As the group effect was significant (*p* < 0.001, 32.5% of total variation), Bonferroni adjustments for multiplicity test were used to compare the group effect for each tissue. A one-way ANOVA was used to compare the histopathological score, ABS and ACS. As the effect of the “treatment” factor was significant for ABS (*p* = 0.0059) and ACS (*p* = 0.0168), a Newman–Keuls test was used to compare the treatment effects between groups. No differences were observed for histopathological score.

## Results

### Early treatment with the low-dose led to rapid and total elimination of *M. haemolytica* in BAL

**Figure 2:**
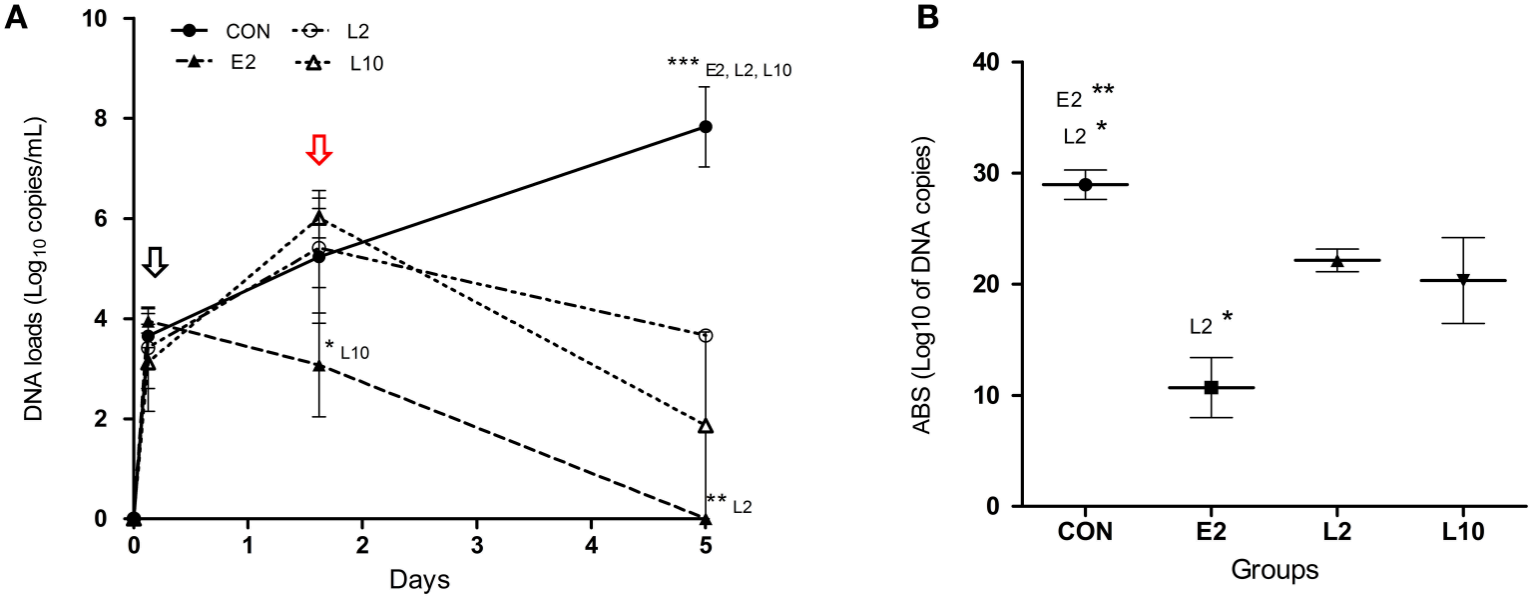
**Mean titers (qPCR, Roche Light Cycler 480, standard 2nd derivative method) of ***M. haemolytica*** DNA copies (with standard deviations) in bronchoalveolar lavages (A) and mean accumulated bacterial shed (B) in calves treated with 2 mg/kg (E2) marbofloxacin at 4 h after inclusion or 2 mg/kg (L2) or 10 mg/kg (L10) marbofloxacin at 36 h after inclusion**. The black and red arrows indicate the time of early and late treatments respectively. Significant differences: ^*^p < 0.05; ^**^p < 0.01; ^***^p < 0.001.

The mean DNA loads of *M. haemolytica* in BAL over time as well as the mean accumulated bacterial shedding (ABS) scores are presented in Figure 2 for three calves of each group (Supplementary Material).

In the CON group, *M. haemolytica* DNA loads increased regularly over time from inclusion (T0) to the end of the experiment (T3) in all the three tested calves, from 3.3 to 8.6 log_10_ DNA copies/mL of BAL, suggesting strong replication of the bacteria in the lower respiratory tract. At T1, just prior to early treatment, bacterial DNA loads were similar between the four groups, at approximately 3 log_10_ DNA copies/mL of BAL. Differences were then observed according to treatment. In the E2 group, marbofloxacin administration at T1 induced a drastic decrease in the bacterial load until euthanasia. At T2, one of the three E2 calves was negative, and all E2 calves were negative at the end of the experiment (T3). In groups L2 and L10, which were treated 33–35 h later, the bacterial load increased in a similar manner as the control group until T2 of late treatment. Then, the bacterial load decreased in both groups, and this decrease appeared to be higher in the L10 group treated with 10 mg/kg marbofloxacin. At T3 of euthanasia, only one L10 calf exhibited positive BAL (5.6 log_10_ DNA copies/mL), whereas all the three L2 calves were positive, with loads ranging from 3.6 to 3.7 log_10_ DNA copies/mL.

The mean *M. haemolytica* DNA loads were significantly different between the CON group and the E2, L2, L10 groups at T3, between E2 and L2 at T3, and between E2 and L10 at T2 (Figure 2A). In addition, ABS scores in BAL indicated significant differences between the CON and the E2 and L2 groups and between the E2 and L2 groups (Figure 2B).

### Distribution and loads of *M. haemolytica* in respiratory tissues showed differences between marbofoxacin treatments

To further investigate the PCR results, we tested all the 22 included calves for the presence of M. *haemolytica* in BAL and in post-mortem tissue samples at T3 of euthanasia (Supplementary Material). The mean DNA loads in BAL were 7, 0, 2.3, and 1 log_10_ DNA copies/mL for calves in the CON, E2, L2, and L10 groups, respectively, with 4 of 4, 0 of 6, 4 of 6, and 1 of 6 positive calves in each respective group.

**Figure 3:**
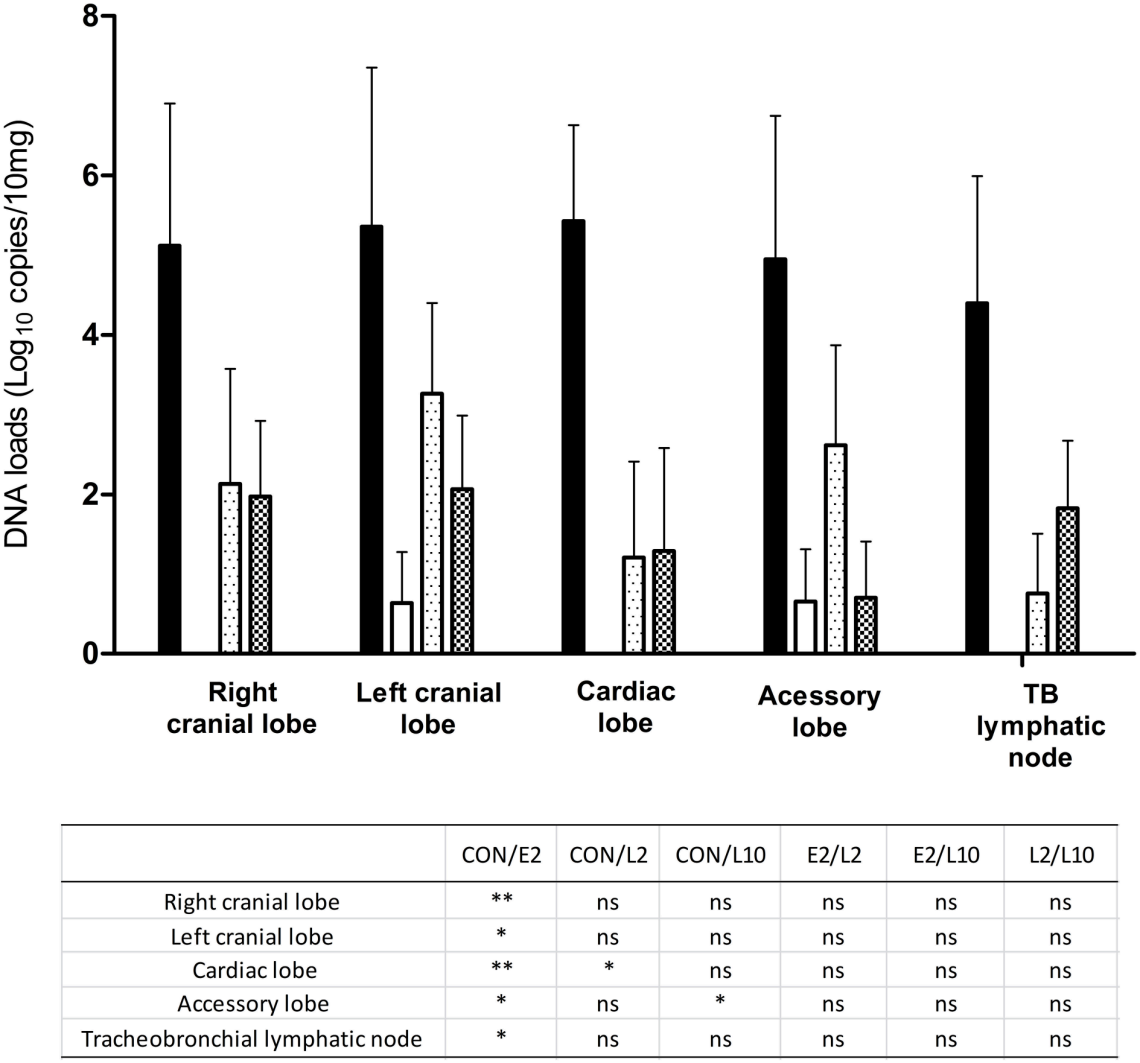
**Mean titers (qPCR, Roche Light Cycler 480, standard 2nd derivative method) of ***M. haemolytica*** DNA copies (with standard deviations) in respiratory post-mortem tissues of calves not treated (
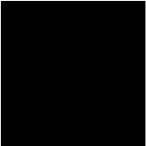
), treated with 2 mg/kg marbofloxacin at 4 h after inclusion (E2, 
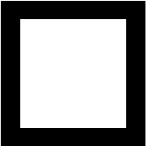
), or 2 mg/kg (L2, 
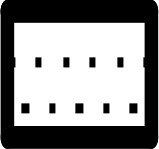
), or 10 mg/kg (L10, 
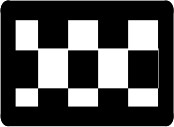
) marbofloxacin at 36 h after inclusion**. Significant differences are shown in the table: ^*^p < 0.05; ^**^p < 0.01.

In parallel, 4 of 4, 2 of 6, 5 of 6, and 4 of 6 calves in the CON, E2, L2, and L10 groups, respectively, were positive for *M. haemolytica* in at least one of the 5 respiratory tissues (right and left cranial lobes, accessory and cardiac lobes, tracheobronchial lymphatic node) tested in each calf. Large differences were observed when considering the number of positive samples per calf and per group. In the E2 group, only the intermediate lobe of one calf and the left cranial lobe of another calf were positive, with moderate titers of 3.8 and 3.9 log_10_ DNA copies/100 mg (Figure 3). In contrast, 80% of the tissue samples of the CON group were positive, whereas the L2 and L10 groups similarly had 37% of tissue samples as positive. In the CON group, high DNA individual titers, ranging between 4.6 and 8.2 log_10_ DNA copies/100 mg, were detected in positive samples (Figure 3). The situation was intermediate in the L2 and L10 groups, with mean loads ranging from 0.7 to 3.2 log_10_ DNA copies/100 mg, depending on the type of tissue. The mean *M. haemolytica* DNA loads in each tissue samples (Figure 3) also indicated significant differences between the CON and E2 group, despite large intra-group variability. To confirm the results obtained by qPCR in tissue samples, a direct bacterial culture and titration was performed on the right cranial lobe of the lungs (Table 1). In the E2 group, all samples collected for bacterial culture remained negative, even the two samples that were slightly positive with PCR. For the CON, L2, and L10 groups, the *M. haemolytica* counts globally mimicked those obtained by PCR, except for two calves in the L2 group for which culture was slightly positive but not with PCR analysis (Table 1).

**Table 1:** **Main individual results of clinical and bacteriological examination after ***M. haemolytica*** challenge for untreated calves (CON) or calves treated with 2 mg/kg (E2) marbofloxacin at 4 h after inclusion or 2 mg/kg (L2) or 10 mg/kg (L10) marbofloxacin at 36 h after inclusion**.

**Treatment group**	**Calf**	**Cranio ventral percentage lung consolidation**	**% of lung lobes with microscopic lesions**	**Highest individual clinical score**	**Accumulated clinical score**	***M. haemolytica* in right cranial lobe (log_10_ CFU/g)**	***M. haemolytica* in right cranial lobe (log_10_ DNA copies/g)**	***M. haemolytica* in BAL at necropsy (log_10_ DNA copies/mL)**
C	4254	88	75	10	34.8	10.5	9.2	8.6
C	4269	0	0	3	11.8	3.0	6.0	7.0
C	4310 euthanized at 56 h	51	87.5	10	24.8	10.0	6.7	7.9
C	4312	12	25	6	20	4.0	7.7	5.5
E2	4253	0	0	3	5.5	0.0	0.0	0.0
E2	4261	0	0	6	9.5	0.0	0.0	0.0
E2	4276	0	0	9	11.5	0.0	0.0	0.0
E2	4284	0	0	3	6.7	.0	0.0	0.0
E2	4298	17	25	5	5.7	0.0	0.0	0.0
E2	4321	0	0	5	8.4	Contamination	0.0	0.0
L2	4263	0	0	8	17.5	3.2	0.0	0.0
L2	4280	55	87.5	12	28.7	8.8	9.4	3.7
L2	4295	0	12.5	6	11.2	0.0	5.3	3.7
L2	4311	11	25	5	9.7	3.7	2.0	4.0
L2	4316	0	0	6	8.4	2.7	2.0	1.2
L2	4319	0	25	8	10.1	0.0	0.0	0.0
L10	4275	0	0	6	10.3	3.2	5.1	5.7
L10	4292	4	37.5	9	14.5	Contamination	4.0	0.0
L10	4306	0	0	10	13.9	0.0	0.0	0.0
L10	4307	0	0	5	8.1	0.0	3.5	0.0
L10	4309	3	25	11	19.9	3.0	5.3	4.1
L10	4313	0	12.5	8	11.9	0.0	0.0	0.0

*Individual clinical scores were calculated as the sum of followings: demeanor from 0 to 3 (normal, dull, depressed, or recumbent); rectal temperature from 0 to 2 (<39.5°C, 39.5–40.5°C, >40.5°C); respiratory rate from 0-3 (<45, 45–60, 60–90, >90); heart rate from 0 to 2 (<80, 80–100, >100); nasal discharge from 0 to 3 (absent, mild, moderate, or profuse); intensity and added lung sounds from 0 to 2 (normal, increased effort, or labored); and appetite from 0 to 2 (normal, decreased, or anorexic). Accumulated clinical scores (ACS) were calculated as the area under daily clinical scores using the trapezoid method. The percentage of cranioventral lung consolidation was calculated as described by Amrine et al. (2014). M. haemolytica quantification was done using qPCR in a Light Cycler 480 (Roche). A standard curve for quantification was obtained by successive 10-fold dilutions of a plasmid containing the same M. haemolytica amplified nucleotide sequence*.

### Treatment with marbofloxacin induce clinical improvement within 24 h

To determine whether bacterial growth was associated with change of clinical status, we assessed calves for clinical signs and lesions over time. All calves were healthy prior to and immediately after inoculation. At this time, the mean rectal temperature was 38.8 ± 0.4°C. Twenty-two of thirty-two calves were selected based on rectal temperature elevation. These calves were randomly allocated into one of four groups just after receiving the respective treatment.

Rectal temperature of the included calves peaked between 3 and 9 h after challenge. Mean rectal temperature peaked at 40.2°C at the time of inclusion in all groups. Then, rectal temperature decreased over time in all groups. Rectal temperature decreased to baseline within 6 h after early treatment in E2 group and within 12 h after late treatment in the L2 and L10 groups. In the non-treated animals, rectal temperature remained above the temperature of the treated groups at each time point, indicating a significant difference between E2 calves and non-treated calves at T2 (data not shown).

**Figure 4:**
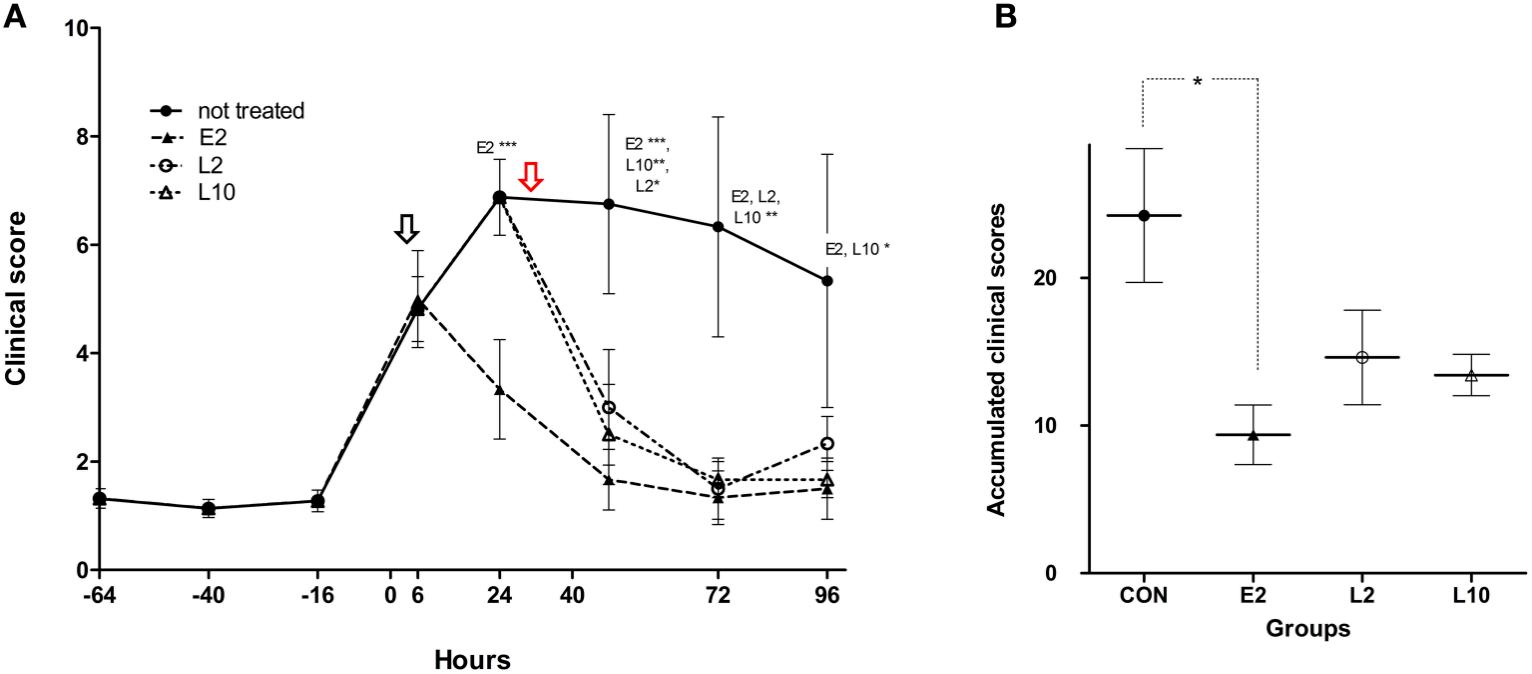
**Mean clinical scores (A) and mean accumulated clinical scores (B) of calves inoculated with ***M. haemolytica*** and treated with 2 mg/kg (E2) marbofloxacin at 4 h after inclusion (black arrow) or 2 mg/kg (L2) or 10 mg/kg (L10) marbofloxacin at 36 h after inclusion (red arrow)**. For **(A)**, calves remained in the control group until the time of treatment. For **(B)**, individual ACS were determined as the area under the curve of clinical scores collected at −16, 6, 24, 40, 72, and 96 h (Graph Pad software, La Jolla, USA). Significant differences: ^*^p < 0.05; ^**^p < 0.01; ^***^p < 0.001.

Clinical signs started at 6 ± 3 h and peaked between 24 and 40 h after inoculation for most calves. After peak, clinical signs of CON calves slightly decreased throughout the course of infection. In the other groups, clinical signs were very similar to those observed in the CON group until each treatment. Consequently, for clinical score evaluation, all calves were considered as a unique group until marbofloxacin administration, as shown in Figure 4A. To summarize, the clinical signs were moderate in most of calves and severe in two CON calves over time and one L2 calf and one L10 calf prior to treatment. In the CON group, one of the two calves with severe disease was euthanized at 56 h after infection for ethical reasons. Moderate signs included hyperthermia, tachypnea and dyspnea with labored breathing. In severe forms, reduced appetite, depression, recumbence, and severe dyspnea with labored breathing and abnormal lung sounds (wheezes and crackles) were observed.

The mean clinical scores indicated that marbofloxacin injections were followed by a rapid (within 24 h) clinical improvement of calves, regardless of early or late treatment (Figure 4A). Twenty-four hours after each respective treatment, 4 of 6 E2 and L2 calves and all of the L10 calves presented a clinical score less than 4 (Table 1). Forty-eight hours after treatment up to the end of the experiment, clinical scores remained < 4 for all calves in the treated groups. Significant differences were observed between the non-treated animals and the E2 group at 24 h, between the CON group and E2, L2, and L10 groups at 48 and 72 h after infection, and finally between the CON group and the E2 and L10 groups at 96 h post infection (Figure 4A).

The accumulated clinical score (ACS) was evaluated for each calf of each group from the time of infection until the time of euthanasia (T3). The mean ACS for each group indicated strong differences between the treated and non-treated groups, with significant differences only between the E2 and CON groups (Figure 4B).

### Early treatment with the low-dose prevent pulmonary lesions

Lung lesions were primarily observed in all calves in the CON group. Lung damage was observed in the cranial lobes and was typical of acute infection with *M. haemolytica*, including a high tissue consolidation and a dark-red to gray-red lobular coloration with foci of necrosis. Lung lesions were also frequently associated with pleuresia and fibrin deposits. The mean percentage of cranioventral lung consolidation was 38, 5, 11, and 1% in the CON, E2, L2, and L10 groups, respectively. However, the extension of the gross lesions varied greatly between animals. In the CON group, two calves exhibited severe lesions on both cranial and caudal lobes, one calf exhibited lesions on cranial lobes only, and one calf presented only very restricted lesions. In all groups, calves with the most severe lung lesions also presented the highest individual clinical score and high *M. haemolytica* content in the lungs (Table 1).

At histopathology, 75, 16.7, 66.7, and 50% of CON, E2, L2, and L10 calves, respectively, had microscopic lesions in at least one lobe of the lung. Considering the extension of the microscopic lesions, 46.9, 4.2, 22.9, and 12.5% of the lobes were positive in the CON, E2, L2, and L10 groups, respectively.

**Figure 5:**
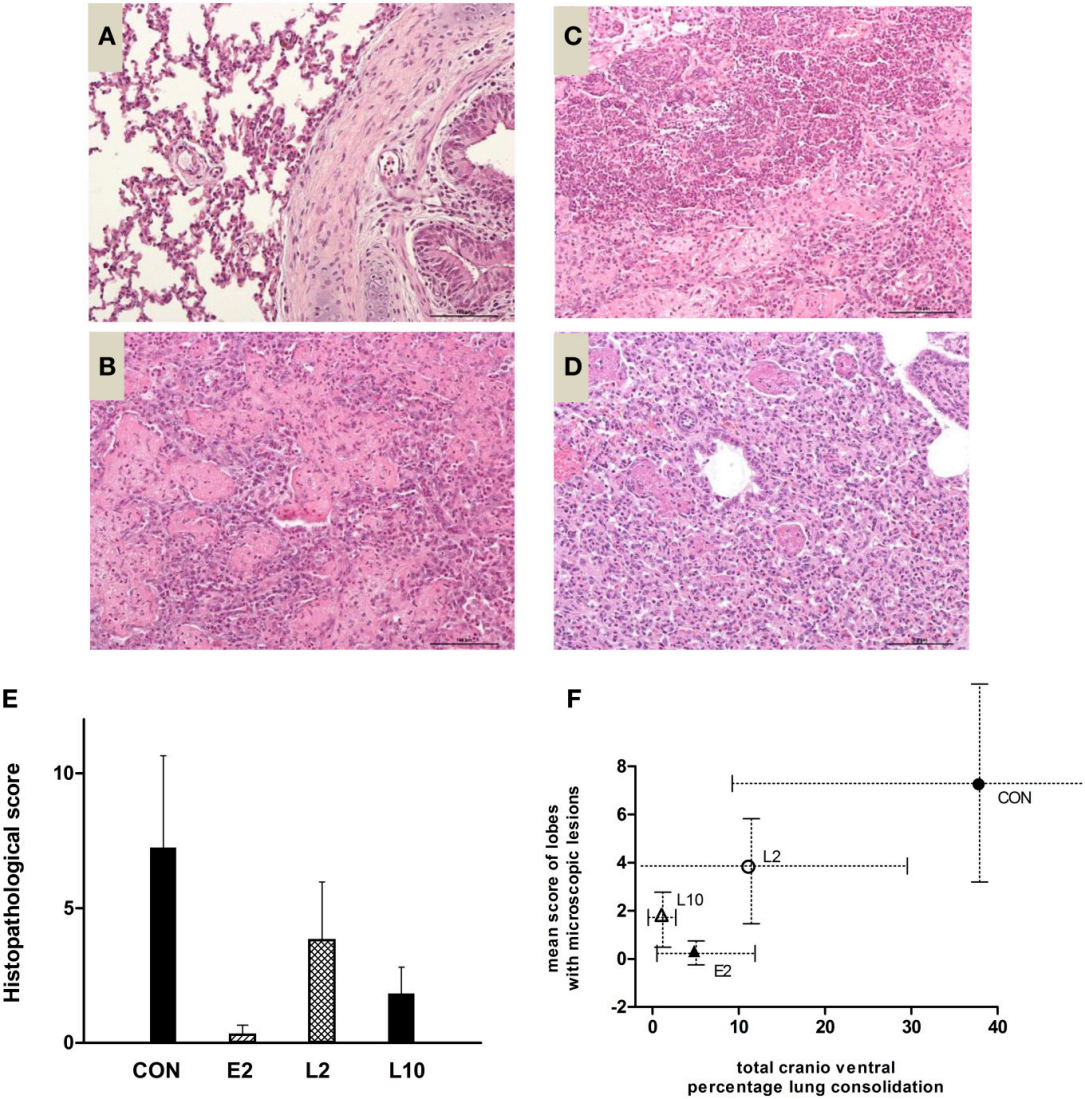
**Microscopic examination. (A)** Normal lung microscopic examination (hemalun and eosin, x200). **(B)** Lesions of hemorrhagic alveolitis, large fibrinous deposits, and major infiltration of leucocytes, score of 2 (hemalun and eosin, × 200). **(C)** oat cells and altered neutrophils, score of 2 (hemalun and eosin, × 200). **(D)** Macrophagic alveolitis, score of 1 (hemalun and eosin, × 200). **(E)** Mean histopathological score (+∕− sd) of calves challenged with *M. haemolytica* and treated with 2 or 10 mg/kg marbofloxacin at the early or late stages of infection. **(F)** Correlation between the total cranioventral percentage of lobe consolidation (gross lesions, × axis) and number of lobes with microscopic lesions (y-axis).

In the CON group, microscopic lesions were located in all lung lobes for two of four calves and only in cranial lobes of the two other animals. By comparison with normal lung microscopic examination (Figure 5A), lesions were specific to an acute severe bronchopneumonia and characterized by an hemorrhagic alveolitis with large fibrinous deposits and major infiltration of leucocytes (Figure 5B), foci of necrosis surrounded by oat cells and altered neutrophils (Figure 5C), interlobular edema and thrombosis (Figure 5C), moderate quantities of mucus in the lumen of bronchioles and leucofibrinous pleuresia (score of 2). This type of lesion (score of 2) was not observed for E2 calves but was observed to a small extent in three of the six calves in the L2 and L10 groups. In addition, histopathological lesions of sub-acute bronchopneumonia (score of 1) were also observed but with less frequency in only one calf in each of the four included groups. In this case, lesions were characterized by a fibrinous and macrophagic alveolitis associated with small abscesses or pyogranulomas of the lung parenchyma (Figure 5D).

The mean histopathological score for each group is shown in Figure 5E, with strong but no significant differences between the treated and not treated calves, except between the E2 and CON groups. This mean score was correlated with the total cranioventral percentage of lobe consolidation (Figure 5F), except for one E2 calf that exhibited a low histopathological score, despite moderate consolidation.

## Discussion

The objective of this study was to assess the efficacy of a decreased marbofloxacin regimen when administered at early-time of infection, on bacterial burden and clinical outcomes in calves infected with *M. haemolytica*. To fulfill this objective, we first developed an experimental model for the detection and treatment of *M. haemolytica* mimicking field conditions. BRD treatments are frequently administered after visual appraisal of clinical respiratory signs. However, because these signs often occur late in the disease course (Schaefer et al., 2007), hyperthermia is increasingly frequently used to promptly detect and treat BRD in young cattle. In a French field study conducted in young bulls, Timsit et al. (2011) demonstrated that episodes of hyperthermia (>39.7°C) preceded the onset of nasal discharge, abnormal lung sounds, and depression by a mean of 19, 38.8, and 51.2 h, respectively. To be consistent with these field observations, we delayed early and late treatments by 30 h. Moreover, we included calves for early treatments based on an increase in rectal temperature of 1°C compared to their basal rectal temperature. This 1°C implementation matches with the temperature threshold commonly used for treatment and previously reported as an early sign of BRD (Galyean et al., 1995; Schaefer et al., 2007; Timsit et al., 2011). At the same time, a challenge dose of 4 × 10^7^ CFU/calf was used to mimic the clinical signs of a natural infection. First, in a previous pilot study, we tested two doses: 10^10^ and 10^7^ CFU/calf using the same model of infection (data not shown). The high inoculum lead to a severe superacute disease inducing death in the first 24 h, as observed in other experimental models using bacterial load infused in lungs over 10^9^ CFU (Fajt et al., 2004; Burciaga-Robles et al., 2010; Hanzlicek et al., 2010; Rose-Dye et al., 2011). In contrast, the 10^7^ CFU dose was associated with a moderate to severe disease evolving in 4–5 days and allowed longer clinical and bacteriological monitoring at the infectious site. Secondly, the qPCR threshold (Ct) values of non-treated calves of these two studies ranged between 19–32 and 19.9–35 in the BAL T2 or T3 and the lung tissues, respectively. These values fit with those obtained (Ct range of 24–36) by the same method for the field BRD diagnostic during a winter season (personal data from 105 samples with 24 samples positive for *M. haemolytica*). Finally the choice of the two doses of marbofloxacin was based on a PK/PD evaluation of antimicrobial efficacy previously published for *M. haemolytica* (Valle et al., 2012; Potter et al., 2013). The high dose of 10 mg/kg matched with the current recommended dose of marbofloxacin, as mentioned in the commercial product (FORCYL®) with the indication for treatment of BRD. The low dose was the lowest dose ensuring bactericidal efficacy *in vitro* on a targeted low inoculum of *M. haemolytica* (Lhermie et al., 2015).

To our knowledge, this is the first study assessing the kinetics of *M. haemolytica* loads in the lower respiratory tract of calves for more than 5 consecutive days. Our results in the control group (Figure 2) indicated a continuous increase in bacterial loads in the BAL from the start to the end of the experiment, confirming our model of infection. Unfortunately it was not possible to strengthen our PCR results by bacterial load counts for practical reasons. Nevertheless, these assessments were performed using a commercialized qPCR kit that is highly repeatable and reproducible and that is increasingly used to diagnose *M. haemolytica* in the field (Pelletier, 2013). PCR or qPCR methods were also shown to detect significantly higher number of positive cases than culture for *M. haemolytica* or *H. somni* (Tegtmeier et al., 2000; Angen et al., 2009; Bell et al., 2014) and much faster (Guenther et al., 2008). To our knowledge, only another study assessed *M. haemolytica* growth during the 48 h after infection with a similar inoculum load (10^8^ CFU/calf). The authors observed an increased bacterial count in bronchial secretions until 18 h post-infection, with a plateau of 10^7^ CFU/mL until the end of the experiment (Sarasola et al., 2002). Differences of bacteria kinetics between the two studies may be due to the method of quantification because part of the DNA for PCR titration may have been obtained from bacteria killed by the immune response of calves. Another possibility is virulence difference of the *M. haemolytica* A1 strain used in this study, which was previously confirmed to be highly replicative and pathogenic in calves.

Our model of *M. haemolytica* infection showed that a single early treatment with a single dose of 2 mg/kg marbofloxacin led to the rapid elimination of bacteria in all infected calves between 30 and 116 h after injection. All E2 calves were negative for *M. haemolytica* detection in BAL at the end of the experiment. Only 2 out of 30 tissues samples contained low DNA quantities (<4 log_10_ copies/100 mg). The rapid clearance of *M. haemolytica* after early treatment with a lower marbofloxacin dose than that currently recommended (10 mg/kg) might be explained by the relatively low loads of bacteria observed in BALs at T1, suggesting that the required regimen to obtain bacterial elimination is dependent (partly or mostly) on the size of the inoculum at the time of treatment. These results are supported by those obtained in various *in vitro* and *in vivo* studies conducted with fluoroquinolones, stressing the influence of bacterial cell density on the efficacy of these antibiotics (Mizunaga et al., 2005; Ferran et al., 2007; Kesteman et al., 2009; Udekwu et al., 2009). Moreover, in a recent experimental study testing bactericidal efficacy of marbofloxacin in a mouse-lung infection model using either *M. haemolytica* or *P. multocida* strains with close marbofloxacin MICs, we observed that the administration of a lower dose of marbofloxacin (given 10 h after inoculation) led to total eradication of a bacterial load of 10^8^ CFU/lung of *M. haemolytica*. In contrast, a similar dose completely failed to achieve bacterial elimination of a bacterial load of 10^8^ CFU/lung of *P. multocida* (Lhermie et al., 2015). These results indicated that marbofloxacin was much more efficacious against *M. haemolytica* than *P. multocida*, in a mouse model which is not a natural host of these pulmonary pathogens. In the natural host model tested in the present study, which is particularly sensitive to the virulence factors expressed by *M. haemolytica* such as leukotoxin (Van Donkersgoed et al., 1993; Apley, 1997; Zecchinon et al., 2005), we confirmed the ability of an early marbofloxacin treatment to produce rapid and complete microbiological cure.

Compared with the early treatment, calves treated 30 h later with the same 2 mg/kg dose of antibiotic displayed a different pattern. Significant differences in DNA titers were found between the E2 and L2 groups in BALs at T3 and in accumulated bacterial loads. Large differences in *M. haemolytica* DNA titers, although not significant, were also observed between the two groups considering both the number of calves with positive lung samples and the total number of positive tissues per calf. The situation appears to be slightly different when calves were treated later but with a high 10 mg/kg dose of marbofloxacin. No significant differences were observed between the E2 and L10 groups at T3 of euthanasia. In the L10 group, *M. haemolytica* was detected in 10 of 30 respiratory samples but in only 2 of 6 tested calves. Further, even if bacterial loads were similar in the L2 and L10 calves just prior to treatment (T2), only one L10 calf presented moderate bacteria DNA titers in BAL 3 days after treatment (T3), whereas all the three L2 calves remained positive, suggesting that an increased marbofloxacin dosage from 2 to 10 mg/kg was associated with higher bacterial elimination when treatments area initiated later. This result is also supported by the decrease in mean DNA titers shown in Figure 2, suggesting that clearance of bacteria was faster for the L10 than L2 group and was likely similar between the L10 and E2 groups. These results are in agreement with previous works, confirming that fluoroquinolones display a concentration-dependent killing mechanism of *M. haemolytica* (Sarasola et al., 2002; Illambas et al., 2013). Finally, our results simultaneously confirm the concentration-dependent behavior of fluoroquinolones against Gram-negative bacteria for a given bacterial load, and suggests that the size of the bacterial load at the time of treatment influences the range of exposure magnitude required to obtain bacterial eradication.

Taken together, these results from an experimental model highlight the benefits of using early treatments with a low dose of antibiotic that should lead to a fast cure of *M. haemolytica* infection in the lower respiratory tract. One major question for livestock professionals is the impact of such early treatment on animal health and also human health by limiting antibiotic consumption. In this study, we only focused on animal health by assessing clinical signs and lesions of infected and treated calves. In the control group, rectal temperature and mean clinical score peaked within 3–12 and 24–40 h following challenge, respectively, reached a plateau, and then slightly decreased until the end of the experiment. This mimics the precedence of hyperthermia as observed in field conditions and supported in numerous respiratory challenge models (Fajt et al., 2004; Burciaga-Robles et al., 2010; Hanzlicek et al., 2010; Forbes et al., 2011; Rose-Dye et al., 2011; Crouch et al., 2012). In our challenge model reproducing moderate to severe disease, early or late treatments with marbofloxacin clearly induced recovery of all calves in <24 h. These results are supported by those obtained in field studies that have monitored the effects of marbofloxacin on rectal temperature and clinical status of animals naturally infected with various respiratory pathogens (Thomas et al., 2001; Grandemange et al., 2012). Total ACS showed major differences between the control and E2, L2, and L10 groups; however, these differences were only significant between the E2 and control groups (Figure 4B). The main explanations may be the variability of individual response in the challenge model or more likely, the delay of treatment, as clinical scores decreased similarly in all treated groups within 24 h after antibiotic injection, with significant differences compared with the control group (Figure 4A).

Our results also suggest that early treatment may prevent pulmonary lesions. Whatever the antibiotic dosage, treatment led to a low frequency of lung lesions, but significant differences for histopathological scores were only observed between the E2 and control groups. Further, calves treated earlier presented a lower percentage of cranioventral lung consolidation, a lower number of lobes with macroscopic and microscopic lesions, and lower histopathological scores compared with calves treated 30 h later. Again, these results could be associated with rapid elimination of bacterial burden, avoiding bacterial growth and thus the onset of pulmonary damage. We also observed that lesion severity was correlated with bacterial load in the lungs. Our results agree with those reported in another experimental BRD model, in which a positive relationship between the severity of clinical signs, cumulative clinical scores, and extension of pulmonary macroscopic and microscopic lesions was observed (Forbes et al., 2011).

However, it is unclear why, despite increasing high qPCR titers in the lungs, we did not observe severe disease with extensive pulmonary lesions in some of the non-treated animals. This observation suggests that implementation of on-field therapy based on early diagnosis of illness and using decreased antimicrobial regimen may require specific diagnosis tools to measure bacterial load and/or, if possible, a precise monitoring of markers correlated with early infection stage.

Promoting a dose modulated concurrently with the infectious bacterial load could be difficult to implement in cattle production under actual field conditions. Indeed, quantification of bacterial content in respiratory samples is not currently performed, although the present qPCR is now used in several veterinary laboratories (Pelletier, 2013). The main caveat is the minimum 1 day-time to obtain a quantitative result, which implies that the treatment would delivered with a delay, when bacterial loads would be already higher than when initially tested. However, constantly more rapid and easier techniques are being developed for detection and quantification of bacteria such as LAMP-PCR, biosensors, reflectance spectroscopy, raman spectroscopy etc….(for review Noble and Weisberg, 2005; Velusamy et al., 2010). Since our model and another study (Sarasola et al., 2002) showed a correlation between the bacterial content and the apparition and evolution of the disease, on-field strategy based on early diagnosis of illness (with efficient markers) and using decreased antimicrobial regimen could be considered in field conditions. We recently performed a field study to evaluate antimicrobial consumption and therapeutic efficacy of an early vs. late treatment protocol with a similar decreased antimicrobial regimen (marbofloxacin 2 or 10 mg/kg) in 195 young bulls with BRD. Our results suggest that the combination of an early detection of disease (increase of body core temperature) with a decreased marbofoxacin treatment reduces drug consumption at the herd level without affecting treatment efficacy compared to a standard therapy. (Lhermie et al., submitted). Such findings evidence the possible rationalization of antimicrobial use under field conditions, to limit impact on public health.

To conclude, to our knowledge, this is the first time that a decreased antimicrobial regimen administered at early time of infection and targeting a low bacterial load demonstrated efficacy in a bovine natural host experimental model. The responses to the treatments, in terms of bacterial elimination, pulmonary lesions, and clinical recovery, were similar between the lower dose (low inoculum-adjusted) administered at an early time of infection and the higher dose (high inoculum-adjusted) given at a later time of infection. Altogether, our results are proof of principle that there is a place for the optimization of antibiotic doses in food-producing animals, provided strict conditions of early diagnosis of illness and/or bacterial load that allow combining the cure of bacterial infections with the reduction of antibiotic consumption. Regarding antimicrobial consumption under public health considerations, further studies evaluating the benefits of modulating dose regimens have to be encouraged.

## Author contributions

All authors listed, approved the work for publication. GL, AB, and GM designed the study; GL, DP, HC, AF, MD, and GM performed the experiments; GL, AF, SA, FG, MS, FW, and GM analyzed the data; GL, AB, and GM wrote the manuscript.

## Funding

This work was supported by the Vetoquinol Company (Lure, France). The funders (Vetoquinol) had no role in study design, data collection and analysis, decision to publish, or preparation of the manuscript.

### Conflict of interest statement

The authors declare that the research was conducted in the absence of any commercial or financial relationships that could be construed as a potential conflict of interest.
