# Supplementary material for: Impact of Timing and Dosage of a Fluoroquinolone Treatment on the Microbiological, Pathological, and Clinical Outcomes of Calves Challenged with Mannheimia haemolytica
Source: Front Microbiol. 2016 Mar 2;7:237. doi: 10.3389/fmicb.2016.00237 (PMC4773444; doi:10.3389/fmicb.2016.00237)
Supplement: Supplementary file 1 [file DataSheet1.pdf]

1- kinetic PCR results (Log_{10}/ml, transformation) from BAL

groups		CON			E2			L2			L10		
	N° calf	4254	4269	4310	4253	4261	4276	4280	4295	4315	4255	4275	4292
Days	Time												
0	T0	0	0	0	0	0	0	0	0	0	0	0	0
0,125	T1	3,53	3,51	3,92	4,39	3,92	3,55	3,65	2,51	4,09	3,79	4,38	1,2
1,62	T2	4,71	6,1	5,89	4,2	4,02	1	6,03	5,68	5,52	6,74	5,91	5,38
5	T3	8,59	6	7,91	0	0	0	3,7	3,7	3,58	0	5,6	0

2- PCR results on BAL (Lo_{g10}/ml) of the 22 calves at euthanasia (T3)

Groups	CON				E2						L2						L10					
calf N°	4254	4269	4310	4312	4253	4261	4276	4284	4298	4321	4280	4295	4296	4311	4315	4316	4255	4275	4292	4307	4313	4318
Log10 copies	8,59	6,00	7,91	5,54	0,00	0,00	0,00	0,00	0,00	0,00	3,70	3,70	2,70	0,00	3,58	0,00	0,00	5,70	0,00	0,00	0,00	0,00

3- PCR on respiratory tissues (Log_{10}/100mg)

	CON-GROUP (4 calves)				E2-group (6 calves)						L2-group (calves)						L10 group (6 calves)					
Right cranial lobe	8,16	0	5,65	6,67	0	0	0	0	0	0	0	8,43	4,34	0	0	0	0	5,09	0	2,47	4,28	0
Left cranial lobe	7,61	0	9,06	4,76	0	0	0	3,83	0	0	0	7,17	0	3,91	4,54	3,94	0	4,18	0	3,94	4,26	0
Cardiac lobe	5,88	2,16	5,7	7,97	0	0	0	0	0	0	0	7,24	0	0	0	0	0	0	0	0	7,75	0
Accessory lobe	7,95	0	7,25	4,6	3,94	0	0	0	0	0	0	7,24	0	4,04	0	4,42	0	0	0	0	4,22	0
TB lynphatic node	6,68	0	6,82	4,09	0	0	0	0	0	0	0	4,52	0	0	0	0	0	4,21	0	0	2,6	4,14
